# Fatty acid stable isotopes add clarity, but also complexity, to tracing energy pathways in aquatic food webs

**DOI:** 10.1002/ecs2.3360

**Published:** 2021-02-07

**Authors:** Ariana M. Chiapella, Martin J. Kainz, Angela L. Strecker

**Affiliations:** ^1^ Department of Environmental Science and Management Portland State University Portland Oregon 97201 USA; ^2^ WasserCluster Lunz—Inter‐University Centre for Aquatic Ecosystem Research Lunz am See A‐3293 Austria; ^3^ Department of Biomedical Research Danube University Krems Krems an der Donau Austria; ^4^ Institute for Watershed Studies Huxley College of the Environment Western Washington University Bellingham Washington 98225 USA; ^5^ Department of Environmental Sciences Huxley College of the Environment Western Washington University Bellingham Washington 98225 USA; ^6^ Present address: Rubenstein School of Environment and Natural Resources University of Vermont Burlington Vermont 05401 USA

**Keywords:** allochthonous subsidies, Arctic Char, compound‐specific stable isotopes, diet tracing, energy flow, fatty acids, lake, mesocosms, trophic ecology

## Abstract

Tracing the flow of dietary energy sources, especially in systems with a high degree of omnivory, is an ongoing challenge in ecology. In aquatic systems, one of the persistent challenges is in differentiating between autochthonous and allochthonous energy sources to top consumers. Bulk carbon stable isotope values of aquatic and terrestrial prey often overlap, making it difficult to delineate dietary energy pathways in food webs with high allochthonous prey subsidies, such as in many northern temperate waterbodies. We conducted a feeding experiment to explore how fatty acid stable isotopes may overcome the challenge of partitioning autochthonous and allochthonous energy pathways in aquatic consumers. We fed hatchery‐reared Arctic Char (*Salvelinus alpinus*) diets of either benthic invertebrates, terrestrial earthworms, or a mixture of both. We then compared how the stable carbon isotopes of fatty acids (δ^13^C_FA_) distinguished between diet items and respective treatments in *S. alpinus* liver and muscle tissues, relative to bulk stable isotopes and fatty acid profiles. Although a high degree of variability of fatty acid stable carbon isotope values was present in all three measures, our results suggest that the ability of this method to overcome the challenges of bulk stable isotopes may be overstated. Finally, our study highlights the importance of further experimental investigation, and consideration of physiological and biochemical processes when employing this emerging method.

## Introduction

A central goal in ecology is to understand the flow of energy within and across ecosystems (Teal 1962, Odum 1968), and theories related to energy flow, trophic dynamics, and cross‐ecosystem interactions continue to evolve. Lakes are an especially important context in which to study these theories, as they are sentinels of environmental change, exist in many different ecotones across the world, and have distinct ecosystem boundaries (Schindler 2009). Although lake food webs are often simplified into two major diet trajectories (benthic and pelagic), a wealth of evidence now supports strong habitat coupling, and significant allochthonous subsidies, especially in small lakes and ponds and northern temperate waterbodies (Schindler and Scheuerell 2002, Vander Zanden and Vadeboncoeur 2002, Pace 2004, Carpenter et al. 2005, Milardi et al. 2015). These phenomena are observed both at the base of the food web (via carbon and nutrient cycling; e.g., Pace 2004, Solomon et al. 2011) and the apex of the food web (via cross‐habitat foraging and terrestrial insect subsidies; e.g., Vander Zanden and Vadeboncoeur 2002, Milardi et al. 2015). Because large fish are often omnivorous and can migrate across habitat boundaries within lakes, accurately tracing and quantifying their energy sources is a challenge. However, partitioning energy sources is critical to understand trophic energy flow and the role of subsidies in lakes. As a new approach to this persistent challenge, compound‐specific stable isotopes may serve as more informative tracers, but their use in freshwater systems is thus far limited (Bec et al. 2011, Boecklen et al. 2011, Larsen et al. 2013).

Traditional research methods on trophic energy flow, such as gut content analysis and bulk stable isotope analysis, can provide insight into energy pathways, but have many assumptions and limitations (Flynn et al. 2018). For instance, gut content analyses provide dietary information for just a snapshot in time, and identification is biased toward organisms that are not easily digested (Grey 2006). Bulk stable isotope analysis estimates trophic position of organisms based on nitrogen stable isotope ratios (δ^15^N) and basal energy sources based on carbon (δ^13^C), and more recently hydrogen (δ^2^H) stable isotope ratios to differentiate aquatic vs. terrestrial basal resources in their tissues. These methods are subject to seasonal variability, discrepancies in turnover time between food web items, and significant error without a strong isotopic baseline (Post 2002, Grey 2006, Brett et al. 2018). In addition, our ability to precisely trace dietary energy along specific pathways is limited because bulk stable isotopes cannot identify specific prey species, only coarse functional groups (Peterson and Fry 1987). Delineating these pathways is especially difficult if energy sources have overlapping isotope values, as is common with aquatic and terrestrial energy sources. For example, pelagic phytoplankton often have overlapping carbon isotope values with terrestrial plants due to the assimilation of terrestrial dissolved inorganic carbon (Solomon et al. 2011), while some benthic invertebrates feed directly on particulate terrestrial organic matter (Cole et al. 2006, Holgerson et al. 2016). Using multiple‐isotope mixing models (e.g., Bayesian mixing models; Moore and Semmens 2008) to also incorporate δ^15^N and δ^2^H can help to alleviate this issue in certain systems; however, the aforementioned challenges of seasonal variability and baseline shifts may still lead to uncertainty. Without employing other methods, bulk stable isotope analysis alone may fail to provide enough information to extract details about the flow of carbon within lake food webs (France 1996, Finlay 2001, Grey 2006).

Dietary fatty acid profiles can differ among both prey species (Lau et al. 2012) and lake habitats (Napolitano 1999, Brett et al. 2009), but the accuracy of fatty acid profiles in tracing diet lack consistency. Preliminary evidence suggests that the δ^13^C values of fatty acids (δ^13^C_FA_) can discriminate between diet sources in aquatic consumers at a finer resolution than traditional bulk stable isotope analysis, and this method has shown promise, especially when used in conjunction with fatty acid profiles (Evershed et al. 2007, Budge et al. 2008, Bec et al. 2011, Taipale et al. 2015).

The five most important polyunsaturated fatty acids (PUFA) for physiological function—linoleic acid (LIN; 18:2n‐6), arachidonic acid (ARA; 20:4n‐6), alpha‐linolenic acid (ALA; 18:3n‐3), eicosapentaenoic acid (EPA; 20:5n‐3), and docosahexaenoic acid (DHA; 22:6n‐3)—may be especially useful in tracing dietary sources. Vertebrates lack the enzymes necessary to synthesize the two essential PUFA, ALA and LIN, which are precursors needed to supplement requirements for ARA (LIN → ARA), SDA (stearidonic acid; 18:4n‐3), EPA, and DHA (ALA → SDA → EPA → DHA). Therefore, these PUFA are mostly obtained through diet, yet serve critical physiological functions for aquatic consumers such as fish, including somatic growth and reproduction (Copeman et al. 2002).

Assuming an aquatic consumer's diet has the appropriate PUFA composition and content to meet its physiological requirements, the δ^13^C values of each PUFA in the consumer can be expected to be similar to the δ^13^C_PUFA_ values of the respective dietary PUFA (Bec et al. 2011, Budge et al. 2016). Deviation of δ^13^C_PUFA_ values between a consumer and its diet can be expected when fractionation occurs as a result of bioconversion of the essential precursors ALA and LIN. Bioconversion of these essential fatty acids is necessary when the dietary PUFA composition does not meet the physiological PUFA requirements of the consumer. This scenario can be assessed by the consumer–diet ratio, or the content of a fatty acid in a consumer (e.g., mg fatty acid per g dry weight) divided by the content of that fatty acid in its diet (see Eq. 1). Consumers with high consumer–diet PUFA ratios are not getting sufficient required dietary PUFA and thus have to convert, via elongation and/or desaturation steps, a precursor fatty acid to produce the required PUFA. Therefore, the δ^13^C value of that PUFA will be depleted relative to its diet due to faster use of the lighter isotope during bioconversion, which occurs during fatty acid synthesis (Ruess and Chamberlain 2010). For example, if DHA is not sufficiently present in a fish's diet, then it must be obtained by converting dietary precursors, such as ALA, SDA, and/or EPA (Murray et al. 2014). These transformations would result in an isotopic depletion of DHA in a consumer relative to the shorter‐chain precursors in its diet, because the ^12^C of the precursor fatty acids is taken up faster than the ^13^C for the synthesis of DHA.

Using δ^13^C_PUFA_ may be useful for distinguishing between terrestrial and aquatic diet sources in fish. DHA and EPA tend to primarily be found in aquatic environments because these PUFA are synthesized by algae, while ALA and LIN are found in both aquatic and terrestrial plants (Sayanova and Napier 2004, Hixson et al. 2015). Fatty acid profiles alone are often not sufficient to distinguish autochthonous vs. allochthonous diet sources for omnivorous freshwater consumers (Hixson et al. 2015), but carbon isotope values of certain PUFA may differ between terrestrial and aquatic diet sources (Evershed et al. 2007, Budge et al. 2008, Bec et al. 2011). The use and recycling of carbon within specific compounds remains largely unexplored, and the use of fatty acid stable isotopes could be a powerful tool to trace dietary pathways of carbon. As such, laboratory‐controlled studies are required to understand how stable carbon isotopes of PUFA change between consumers and their diets.

We conducted a laboratory feeding experiment to investigate whether δ^13^C_PUFA_ can distinguish between autochthonous and allochthonous energy pathways more effectively than traditional methods in a simulated low‐productivity food web (i.e., diet‐limited, such as the case in oligotrophic aquatic ecosystems). Juvenile Arctic Char (*Salvelinus alpinus*) were reared in mesocosms to experimentally test the ability of fatty acid stable isotopes to discriminate between terrestrial and benthic dietary sources in an aquatic consumer. Specifically, we tested (1) how δ^13^C_PUFA_ values differ between benthic and terrestrial invertebrates, and (2) how such differences propagate to the δ^13^C_PUFA_ values in *S. alpinus* with benthic, terrestrial, and mixed‐diet treatments. We examined these objectives by feeding fish a diet of either benthic stream invertebrates, terrestrial earthworms, a mix of both, or hatchery‐formulated pellets. We used benthic invertebrates as our aquatic prey because they often feed on terrestrial organic matter, which can lead to overlapping bulk stable isotope values with terrestrial insects (France 1996). Fatty acid profiles, δ^13^C_PUFA_ values, and bulk δ^13^C values of each fish were then analyzed after four weeks and compared to their diets. We expected to observe significant differences in δ^13^C_PUFA_ values between diet sources, and differences in δ^13^C values of ALA, LIN, and EPA in fish with different diet treatments. Additionally, we expected to observe larger differences in δ^13^C_PUFA_ between treatments relative to fatty acid profiles and bulk δ^13^C values (Evershed et al. 2007, Budge et al. 2008, Bec et al. 2011).

## Methods

### Study design

**Fig. 1:**
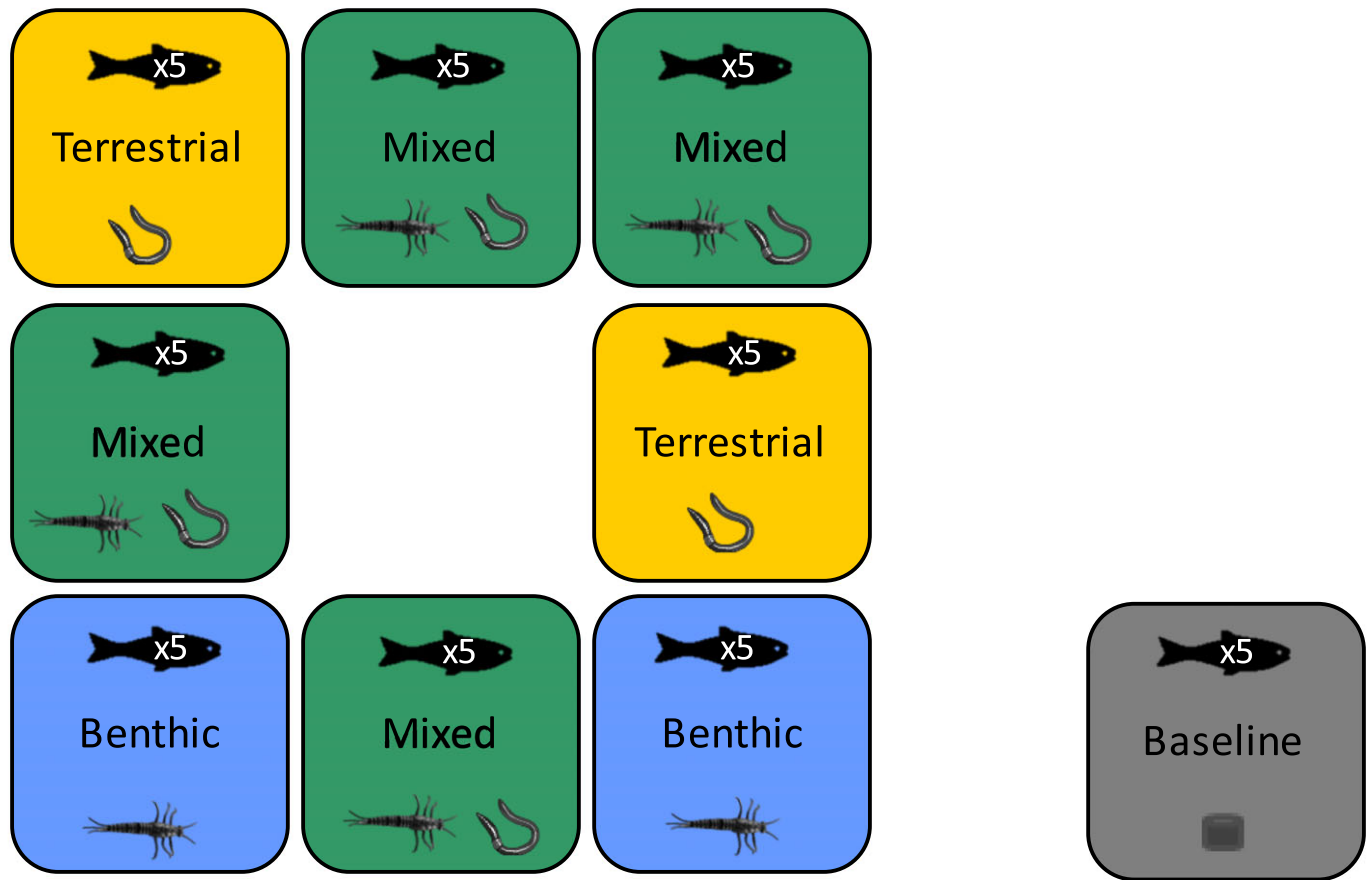
The study included two replicates of the benthic diet treatment, two replicates of the terrestrial diet treatment, and four replicates of the mixed‐diet treatment. Treatments were randomly applied to eight aquaculture tanks, each containing five *Salvelinus alpinus*. Fish in a neighboring aquaculture production tank were continued on pellet feed as part of the normal aquaculture rearing process; five fish were sampled from this tank and analyzed to establish a baseline to compare the treatments against.

We used eight aquaculture tanks (1 m^3^ each) to simulate three diet scenarios: benthic, terrestrial, and mixed (Fig. 1). We also sampled fish that were maintained on a pellet diet as part of an associated aquaculture production to compare with the diet treatments and ensure a change was detected; we refer to these fish as having a baseline diet. Tanks were made of a fiberglass polyester mix, and each tank had an isolated flow‐through system with a continuous supply of gravel‐filtered spring water, which remained between 7.5°C and 9.3°C throughout the experiment. Wastewater was drained using a sinkhole covered by a 5‐mm mesh screen in each individual tank (Murray et al. 2014). Prior to the experiment, we reared *S. alpinus* in a hatchery at WasserCluster Lunz, Austria. We used *S. alpinus* because they are the native apex predator of Lake Lunz and its surrounding streams from which we collected our diet treatments. During hatchery rearing, fish were fed a daily isocaloric pellet diet (Appendix S1: Table S1). We then randomly selected 45 juvenile fish (mean weight 82 g ± 17 SD) to add to the treatment tanks. Five fish were added to each of eight treatment tanks, which were randomly assigned one of the following diet treatments: benthic diet (*n* = 2), terrestrial diet (*n* = 2), mixed benthic, and terrestrial diet (*n* = 4). Additionally, fish in a ninth tank were continued to be fed a pellet diet as part of an aquaculture operation. These fish were used as a reference compared with the treatments, which we refer to as our baseline. Fin clips were used to differentiate fish in each tank. We assigned a higher number of mixed‐diet replicates relative to single‐diet replicates because one of our main objectives was to partition energy sources in a mixed‐diet scenario and we were constrained by the number of tanks available. The experiment was run for four weeks, which we anticipated to be long enough for the new diet to be detected in tissues (Heady and Moore 2013, Mohan et al. 2016). We selected this time frame to mimic how dietary shifts would be detected in nature.

All tanks were supplied with the same dietary biomass daily so that all fish received ~1.5% of their body weight as daily food supply (as in Murray et al. 2014). For the benthic diet treatment, a mix of mayflies, stoneflies, and caddisflies were collected from a neighboring stream each day (Appendix S1: Table S2). Benthic invertebrate functional groups included collectors, scrapers, shredders, and predators. For the terrestrial diet treatment, earthworms (Lumbricidae) were collected from upland riparian soil daily; although the soil was not entirely independent of the riverine system, earthworms primarily process terrestrial material (Curry and Schmidt 2007) and were a more abundant and digestible food source for fish than other terrestrial invertebrate subsidies (such as arthropods). All fresh invertebrates were weighed and portioned out for each treatment tank. The mixed‐diet treatment was composed of a 50:50 biomass mix of benthic invertebrates:earthworms. After weighing, the invertebrates were placed in their respective tanks as fish feed, and tanks were observed until all invertebrates were consumed. Each week, additional samples of benthic invertebrates and earthworms were collected for analysis, as a means of tracking the baseline fatty acid content (i.e., mg fatty acid per g dry weight) and δ^13^C_FA_ values throughout the course of the experiment. After four weeks, all fish were euthanized following the Federal Act on the Protection of Animals, Austria (http://www.ris.bka.gv.at), then dissected for muscle and liver tissues and frozen at −80°C until processing.

### Sample processing and analysis

All sample analyses were completed at WasserCluster Lunz. Fish muscle and liver, benthic invertebrates, earthworms, and pellets were lyophilized and subsequently homogenized using a mortar and pestle. Both fish muscle and liver were analyzed because, while muscle tissue is more representative of long‐term isotope values, liver has a shorter turnover time and is more likely to reflect recently acquired dietary isotope values. Lipids were extracted from each sample using a chloroform:methanol:water (4:2:1) mixture (Heissenberger et al. 2010), with subsequent sonication and vortexing. Fatty acid methyl esters (FAME) were formed by adding toluene and a sulfuric acid‐methanol solution to the lipid extracts. Samples were then kept at 50°C for 16 h. Final extractions for FAME were completed with hexane and centrifugation to release CO_2_, followed by drying with N_2_ and dissolving in hexane. The final FAME extracts were analyzed with a gas chromatograph (GC) equipped with a temperature‐programmable injector and autosampler, and separated using a Supelco^®^ SP‐2560 GC‐column (100 m × 0.2 µm × 0.25 mm, Supelco, Bellefonte, Pennsylvania, USA). Fatty acid content (i.e., mg fatty acid per g dry weight) was calculated using calibration curves based on standard concentrations. Samples were then analyzed for δ^13^C of individual fatty acids using a GC‐isotope ratio mass spectrometer (IRMS) equipped with a Supelco SP‐2560 GC‐column (100 m × 0.2 µm × 0.25 mm), and quantified using internal fatty acid standards.

Compound‐specific stable isotope analyses were used to assess the origin and isotopic carbon (δ^13^C) composition of different FA molecules. Fatty acids were separated using a gas chromatograph (Trace^™^ 1310 Thermo Scientific, Rodano‐Milan, Italy) equipped with a Supelco^®^ SP‐2560 GC‐column (100 m × 0.2 µm × 0.25 mm) with helium as the carrier gas. The GC was coupled to an Isolink 2 where the separated FA were combusted at 1000°C. The generated CO_2_ was transported with the carrier gas (helium) to the Conflo IV where each sample was diluted with the carrier gas helium and connected with the reference CO_2_ gas. Finally, all CO_2_ molecules were analyzed in an isotope ratio mass spectrometer (IRMS; Delta V Advantage; see Kühmayer et al. 2020 for details).

### Data analyses

We compared fatty acid content, and δ^13^C_PUFA_ and bulk δ^13^C values among diet items (i.e., earthworms, macroinvertebrates, pellets) and across treatments (i.e., terrestrial, benthic, mixed) in *S. alpinus* muscle and liver (only of fish that gained weight during the experiment). We used ANOVA linear mixed‐effects models and calculated pseudo‐marginal (fixed factors only) and conditional (fixed and random factors) *R* ^2^ to account for the unbalanced sample size and the random effects of time (for diet items) and tank (for fish). This was followed by a pairwise Tukey‐adjusted least‐squares means (LSM) difference test to compare values across treatments. In the mixed‐effects models for the fish, we used fish as the sample unit, treatment as a fixed factor, and tank as a random factor. To control for the effects of tank (for fish) and time (for diet items), we then calculated the LSM of the fatty acid content and δ^13^C_PUFA_ values for each treatment group. In the mixed‐effects models for the diet items, we used weekly subsamples as the sample unit, treatment as the fixed factor, and time as a random effect, to account for any basal resource shifts throughout the field sampling period. We then conducted a post hoc analysis by calculating the pairwise Tukey‐adjusted differences between the LSM of fatty acid content and δ^13^C values for each treatment to determine whether treatments were significantly different (α = 0.05).

Principal components analysis (PCA) was used to examine differences in fatty acid compositions between diets and among treatments in *S. alpinus* muscle and fish liver. We then fit vectors of major fatty acid groups—saturated fatty acids (SAFA), monounsaturated fatty acids (MUFA), and PUFA—and individual PUFA onto the PCA to determine which fatty acid groups and PUFA were driving differences in fatty acid profiles between diets and between treatment groups.

We calculated consumer–diet ratios (C‐D; also called calibration coefficients; Iverson et al. 2004) for each PUFA by determining the ratio of each PUFA mass fraction (PUFA_C‐D_; mg/g) between fish that gained weight (PUFA_f_) and their respective diets (PUFA_d_):

(1) $${\text{PUFA}}_{\text{C-D}}={\text{PUFA}}_{f}/{\text{PUFA}}_{d}.$$

A PUFA_C‐D_ > 1 suggests that the consumer retained more of this PUFA than directly supplied by its diet. This could be the result of selective dietary PUFA retention and/or enzymatic conversion of precursor fatty acids to this target PUFA, for example, via retro‐conversion as suggested for invertebrates (von Elert 2002). Such conversion may result in isotopically lighter δ^13^C values in the consumer compared with the dietary precursor. Therefore, a high consumer–diet ratio would result in marked differences in δ^13^C values of a particular PUFA between a fish and its dietary precursor fatty acid. We then determined the reliability of each δ^13^C_PUFA_ value as a dietary tracer by calculating the difference in δ^13^C of each fatty acid (Δ ^13^C_FA_) between each fish (δ^13^C_f_) and its respective diet (δ^13^C_d_), and compared this value to the Δ ^13^C_FA_ for precursors ALA and LIN between baseline fish and pellets (i.e., trophic discrimination factor):

(2) $$\Delta^{13}C_{\text{FA}}=\delta^{13}C_{f}‐\delta^{13}C_{d}.$$

All statistical methods were completed using the packages MASS (Ripley et al. 2019), lme4 (Bates et al. 2014), lmerTest (Kuznetsova et al. 2019), lsmeans (Lenth 2018), and MuMIn (Bartoń 2019) in R Studio version 1.1.463 (R Core Team 2016).

## Results

Two fish experienced mortality after approximately 10 d—one from each benthic treatment tank—seemingly due to gas bubble disease (a common phenomenon in hatchery experiments due to oxygen supersaturation from water inflow), resulting in a 5% mortality rate. No other fish showed symptoms of this disease; however, 34 fish lost weight during the experiment due to competition for available food. To avoid the influence of starvation‐induced lipid metabolism, we only included fish that gained weight over the course of the experiment in our analyses (*n* = 23; this did not affect the number of tank replicates).

### Fatty acid content

**Fig. 2:**
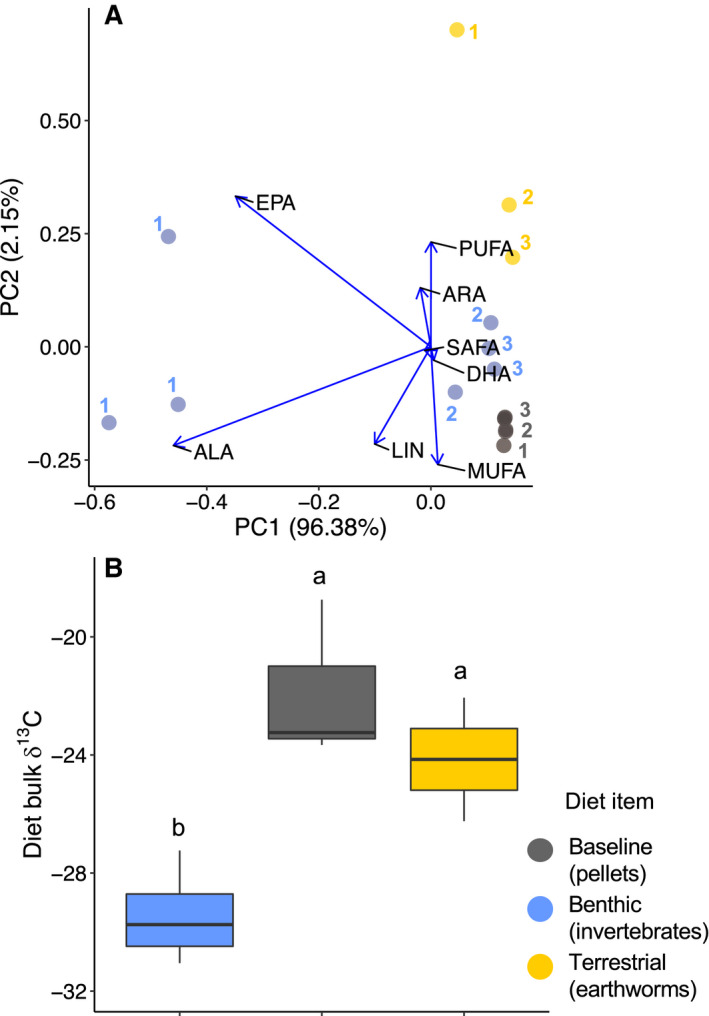
(A) Profiles of major fatty acid groups and individual polyunsaturated fatty acids (PUFA) in diets, as illustrated by principal components analysis. Major fatty acid groups include total saturated fatty acids (SAFA), total monounsaturated fatty acids (MUFA), and total polyunsaturated fatty acids. Arrows show which fatty acid groups or individual PUFA are driving differences in fatty acid profiles between each diet item. The blue dots represent benthic invertebrates, the yellow dots represent earthworms, and the gray dots represent pellets. Number labels indicate week of the experiment during which the diet subsample was taken. (B) Box and whisker plots of mean bulk δ^13^C (‰) in diets. The boxes represent the interquartile range, lines represent the group median, whiskers represent 1.5 times the interquartile range, and dots represent outliers. Letters denote significant difference at *P* < 0.05 (determined using Tukey‐adjusted differences in least‐square means).

We compared differences in fatty acid profiles using a PCA with each fatty acid group fitted as a vector. Fatty acid content in diets and fish varied widely, especially for PUFA (Appendix S1: Table S3). Differences in fatty acid profiles between diets were largely driven by differences in ALA and ARA content, with some differences in LIN, EPA, and DHA (Table 1, Fig. 2). We determined if PUFA content was significantly different between treatments with a mixed‐effects ANOVA, then used a pairwise post hoc test of Tukey‐adjusted LSM differences to determine which treatment combinations were significantly different from each other (Table 1). The ALA content was significantly different among diets (ANOVA *F*_2,13_ = 3.77, *P* = 0.05): Benthic invertebrates had significantly higher ALA than pellets. ARA content was significantly different among diets (ANOVA *F*_2,12_ = 8.72, *P* < 0.01), with higher ARA in benthic invertebrates and earthworms relative to pellets. Benthic invertebrates had higher LIN relative to earthworms, but these differences were not statistically significant among diets (ANOVA *F*_2,11_ = 3.46, *P* = 0.07). EPA was higher in benthic invertebrates relative to pellets, but again, content was not significantly different among diets (ANOVA *F*_2,13_ = 2.95, *P* = 0.09). DHA was somewhat higher in pellets relative to earthworms, but overall was not significantly different among diets (ANOVA *F*_2,13_ = 3.54, *P* = 0.06).

**Table 1:** Pairwise post hoc test *P*‐values for Tukey‐adjusted LSM differences in fatty acid content between diets and between treatments in fish muscle and liver (for fish with weight gain only); bold indicates a significant difference at *P* < 0.05.

Diet treatment	Diets		Muscle			Liver		
	Benthic	Terrestrial	Benthic	Terrestrial	Mixed	Benthic	Terrestrial	Mixed
ALA								
Baseline	(+) **0.03**	0.97	(−) 0.06	0.16	0.26	0.12	0.53	0.85
Benthic		(−) 0.06		0.48	0.18		(+) **0.03**	(−) 0.06
Terrestrial					0.45			0.30
LIN								
Baseline	(+) 0.07	0.93	(−) **0.05**	0.19	0.30	0.28	0.20	0.53
Benthic		(−) **0.04**		0.38	0.12		(+) **0.02**	(−) 0.06
Terrestrial					0.38			0.27
ARA								
Baseline	(+) **<0.01**	(+) **<0.01**	0.20	0.50	0.80	0.20	0.20	0.20
Benthic		0.82		0.50	0.20		0.70	0.60
Terrestrial					−0.50			0.90
EPA								
Baseline	(+) **0.03**	0.62	0.30	0.40	0.80	(+) 0.06	0.84	0.17
Benthic		0.19		0.70	0.30		(+) 0.07	0.27
Terrestrial					0.40			0.22
DHA								
Baseline	(−) 0.09	(−) **0.03**	0.30	0.50	0.70	0.50	0.20	0.40
Benthic		0.28		0.60	0.20		0.60	1.00
Terrestrial					0.20			0.40

Notes

LSM, least‐squares means. If denoted with a (+), treatments along column headers had greater fatty acid content than treatments at row headers; if denoted with a (−), the column header treatment has less fatty acid content (e.g., earthworms had less LIN than benthic invertebrates). Direction of relationship also given for pairs with *P*‐values between 0.05 and 0.10.

**Fig. 3:**
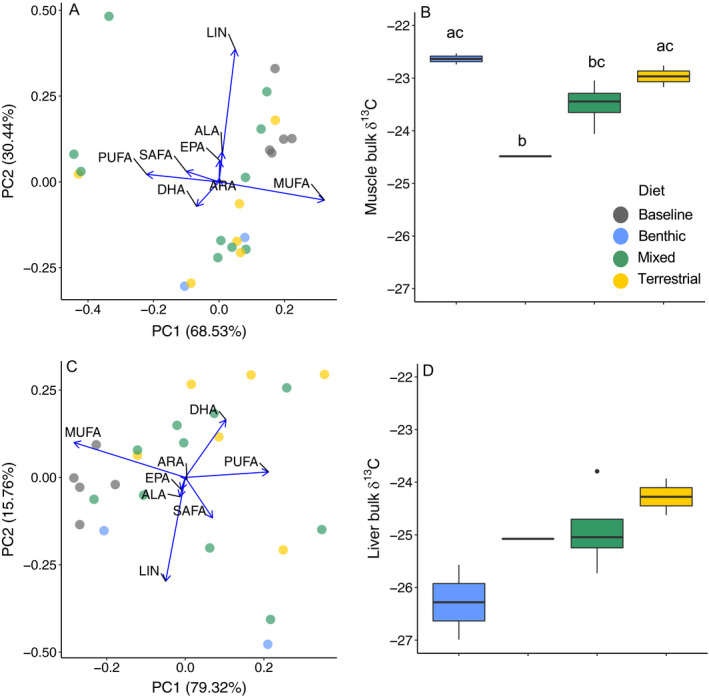
Left panels show profiles of major fatty acid groups and polyunsaturated fatty acids (PUFA) in *Salvelinus alpinus* (A) muscle and (B) liver (for fish with weight gain only), as illustrated by PCA. Arrows show which fatty acid groups or PUFA are driving differences in profiles between diets. The blue dots represent fish given a benthic diet, the yellow dots represent a terrestrial diet, green dots represent a mixed diet, and the gray dots represent a baseline diet. Left panels show box and whisker plots of mean bulk δ^13^C (‰) in *S. alpinus* (C) muscle and (D) liver. The boxes represent the interquartile range, lines represent the group median, whiskers represent 1.5 times the interquartile range, and dots represent outliers. Letters denote in panel (B) significant difference at *P* < 0.05 (determined using Tukey‐adjusted least‐squares means differences); treatments in panel (D) were not significantly different.

Differences in fatty acid content for each fatty acid were not as pronounced in the respective treatments in fish muscle and liver relative to the differences observed in their diets (Table 1, Fig. 3). Fatty acid content (corrected with least‐square means) did not differ significantly among treatments in fish muscle (ANOVA *P* > 0.05 for all models). Consumer–diet ratios of PUFA were low, with the exception of DHA (Table 2). DHA consumer–diet ratios were high for all diet treatments, but most pronounced for fish with terrestrial and mixed diets.

**Table 2:** Consumer–diet ratios of each PUFA for fish liver and (for fish with weight gain only), calculated using Equation 1

Diet treatment	Tissue	Consumer–diet PUFA ratio				
		LIN	ALA	ARA	EPA	DHA
Benthic	Liver	0.87	0.06	0.96	0.11	**15.81**
	Muscle	0.29	0.02	0.20	0.04	**8.97**
Terrestrial	Liver	**3.22**	**1.23**	0.59	0.24	**99.71**
	Muscle	**4.16**	**1.66**	0.15	0.15	**57.81**
Baseline	Liver	**1.60**	**1.36**	**3.71**	**1.69**	**5.51**
	Muscle	**1.60**	**1.36**	**3.71**	**1.69**	**5.51**
Mixed	Liver	0.90	0.07	0.70	0.13	**27.22**
	Muscle	**1.10**	0.08	0.20	0.09	**20.33**

Note

PUFA, polyunsaturated fatty acids. Fatty acids with large (>1) factors are bolded.

### Stable isotopes

**Fig. 4:**
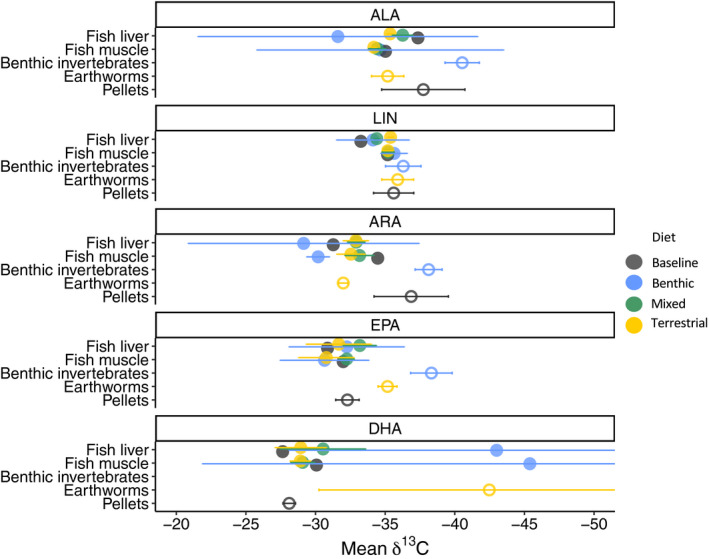
Mean polyunsaturated fatty acids δ^13^C (‰) in fish liver and muscle (for fish with weight gain only; closed circles), and diets (open circles: benthic invertebrates *n* = 6, earthworms *n* = 3, and pellets *n* = 6). Error bars represent standard deviations.

We determined how δ^13^C_PUFA_ values differed among groups using ANOVA, followed by a pairwise post hoc test of Tukey‐adjusted LSM differences to determine which treatment combinations were significantly different from each other (Table 3). Although substantial variability was observed in the δ^13^C_PUFA_, δ^13^C values were significantly different among diets for ALA (ANOVA *F*_2,10_ = 10.66, *P* < 0.01), ARA (ANOVA *F*_2,10_ = 19.74, *P* < 0.01), and EPA (ANOVA *F*_2,8_ = 22.02, *P* < 0.01), but not LIN (ANOVA *F*_2,10_ = 0.32; *P* = 0.73) or DHA (no values for benthic invertebrates; Table 3, Fig. 4). Earthworms were significantly more enriched in δ^13^C_ALA_ (+5‰), δ^13^C_ARA_ (+6‰), and δ^13^C_EPA_ (+3‰) relative to benthic invertebrates. Only traces of DHA were detected in benthic invertebrates (1.22 ± 0.77 mg/g), and no reliable δ^13^C_DHA_ values could be measured (δ^13^C_DHA_ values in earthworms ranged between −33.52‰ and −56.42‰). Pellets had significantly more depleted δ^13^C_ARA_ than earthworms, significantly more enriched δ^13^C_EPA_ than earthworms and benthic invertebrates, and significantly more enriched δ^13^C_DHA_ than earthworms, but otherwise no significant differences existed between treatment and baseline diets.

**Table 3:** PUFA Δ^13^C for each diet treatment in fish muscle and fish (for fish with weight gain only).

Diet treatment	Tissue	Δ^13^C_LIN_	Δ^13^C_ALA_	Δ^13^C_ARA_	Δ^13^C_EPA_	Δ^13^C_DHA_
Benthic	Liver	1.4‰ 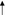	5.4‰ 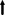	6.2‰ 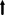	5.5‰ 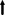	NA
	Muscle	1.1‰ 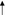	5.2‰ 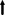	5.4‰ 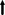	6.0‰ 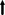	NA
Terrestrial	Liver	1.0‰ 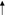	−0.2‰ 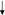	−0.6‰ 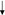	4.3‰ 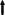	14.7‰ 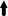
	Muscle	0.8‰ 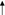	1.1‰ 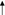	−0.6‰ 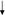	3.9‰ 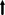	13.4‰ 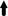
Baseline	Liver	2.4‰ 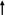	0.4‰ 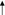	5.6‰ 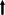	1.4‰ 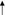	0.5‰ 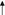
	Muscle	0.4‰ 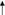	2.7‰ 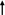	2.4‰ 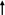	0.3‰ 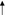	−2.0‰ 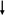

Notes

PUFA, polyunsaturated fatty acids. A positive number indicates the fish tissue is more enriched in δ^13^C_FA_ than its diet; the larger the number, the larger the isotopic fractionation. Upward arrows indicate enrichment of δ^13^C_FA_ in tissue relative to diet, while downward arrows indicate depletion of δ^13^C_FA_ in tissue relative to diet; boldness indicates the size of departure from zero. We did not calculate Δ^13^C values for mixed treatments because we could not assume that fish were getting an exact 50:50 benthic:terrestrial mix due to selective feeding and competition between fish. NA indicates data were not available for one or more group.

The values of δ^13^C_ALA_, δ^13^C_ARA_, and δ^13^C_EPA_ in *S. alpinus* tissues differed from those in their diets (Table 3, Fig. 4). No significant differences in δ^13^C values of fatty acids existed among treatments in fish muscle and fish liver, with the exception of δ^13^C_LIN_ in liver (ANOVA *P* > 0.05 for all models). The patterns exhibited in fish liver δ^13^C_ALA_ and δ^13^C_EPA_ were similar to, but a much smaller magnitude than those seen in their treatment diets (Fig. 4), were not significant at α = 0.05, and had high Δ^13^C values (Table 4). In fact, no significant differences in δ^13^C_ALA_ and δ^13^C_EPA_ were observed among treatments in liver or muscle tissues (Table 4). In some cases, for example with δ^13^C_ARA_, the values exhibited in fish muscle and fish liver were opposite those seen in diets, with the benthic treatment having similar or more depleted δ^13^C_ARA_ relative to the terrestrial treatment (Fig. 4). In fish liver, δ^13^C_LIN_ in terrestrial‐based diets was significantly lower than in fish with mixed and baseline diets (ANOVA *F*_3,19_ = 5.01, *P* = 0.01), even though there were no differences in dietary δ^13^C_LIN_ (Table 4). The random effect of tank explained substantial variability between treatments for δ^13^C_ALA,_ δ^13^C_ARA,_ and δ^13^C_DHA_ (variance explained by tank, 11.36–149.37), but was otherwise small or not present for the other PUFA (Appendix S1: Table S5).

**Table 4:** Pairwise post hoc test for Tukey‐adjusted LSM differences in δ^13^C between diets and between treatments in fish muscle and liver (for fish with weight gain only); *P*‐values are shown values are shown, where bold indicates significant difference at *P* < 0.05.

Diet treatment	Diets		Muscle			Liver		
	Benthic	Terrestrial	Benthic	Terrestrial	Mixed	Benthic	Terrestrial	Mixed
δ^13^C_ALA_								
Baseline	(+) **0.04**	(+) 0.10	0.90	0.90	0.90	(+) 0.10	0.40	0.70
Benthic		(+) **<0.01**		0.90	0.90		0.30	0.10
Terrestrial					0.90			0.60
δ^13^C_LIN_								
Baseline	0.50	0.80	0.50	1.00	1.00	0.27	(−) **<0.01**	(−) **0.04**
Benthic		0.70		0.50	0.50		0.11	0.72
Terrestrial					1.00			(+) **0.05**
δ^13^C_ARA_								
Baseline	0.23	(+) **<0.01**	(+) **0.01**	(+) 0.09	0.20	0.60	0.70	0.70
Benthic		(+) **<0.01**		0.12	(−) **0.04**		0.30	0.30
Terrestrial					0.51			1.00
δ^13^C_EPA_								
Baseline	(+) **<0.01**	(−) **0.02**	0.50	0.50	0.90	0.60	0.70	0.30
Benthic		(+) **0.01**		0.90	0.30		0.80	0.70
Terrestrial					0.30			0.40
δ^13^C_DHA_								
Baseline	NA	(−) **0.01**	0.30	0.90	0.90	(−) 0.10	0.90	0.70
Benthic		NA		0.20	0.10		(−) 0.10	(+) 0.10
Terrestrial					1.00			0.80

Notes

LSM, least‐squares means. If denoted with a (+), treatments along column headers had greater fatty acid content than treatments at row headers; if denoted with a (−), the column header treatment has less fatty acid content (e.g., benthic invertebrates had a lower δ^13^C_ALA_ than pellets). Direction of relationship also given for pairs with *P*‐values approaching significance (*P* < 0.10). NA indicates data were not available for one or more group.

Diets had significant differences in bulk δ^13^C (ANOVA *F*_2,6_ = 9.87, *P* = 0.01; Fig. 2b). Pellets were significantly more enriched in δ^13^C than benthic invertebrates, as were earthworms (Table 4). The differences in bulk δ^13^C values between pellets and benthic invertebrates were not reflected in fish muscle. In fact, the relationship of bulk δ^13^C values between benthic and terrestrial treatments in fish muscle was opposite that seen between the diets (Table 4, Figs. 2b, 3d). Fish with the baseline diet had more depleted bulk δ^13^C values relative to fish with benthic and terrestrial diet treatments. No significant differences in bulk δ^13^C values existed between diet treatments in *S. alpinus* liver, although similar to diets, benthic treatments were more depleted in bulk δ^13^C relative to terrestrial treatments (Table 4, Fig. 3c).

## Discussion

This feeding experiment provides evidence that carbon stable isotopes of PUFA may be another useful diet tracer, but only if the ecosystem context and organism physiology of consumers are well understood. As expected, the δ^13^C values of ALA and EPA differed significantly among earthworms, benthic invertebrates, and pellets. However, our hypothesis that differences in dietary δ^13^C values of precursor fatty acids would propagate in fish tissues was not supported; the δ^13^C values of ALA and EPA in fish muscle and liver were not similar to the δ^13^C_ALA_ or δ^13^C_EPA_ in their respective diet treatments. Contrary to our expectation, the δ^13^C values of precursor LIN were not distinguishable among diet sources. Fatty acid content and bulk δ^13^C values were insufficient in distinguishing between benthic and terrestrial dietary sources in fish; however, the dietary end members had different bulk δ^13^C values. Our data suggest that using δ^13^C_PUFA_ may be an effective diet‐tracing approach for fishes, but metabolic lipid and fatty acid processes in fish must be considered.

We observed differences in fatty acid content among the different diets. Earthworm fatty acid profiles were distinguished by higher PUFA content, which is not typical of terrestrial invertebrates, but some earthworms may obtain EPA and DHA from their gut microbiota (Sampedro et al. 2006). Additionally, the observed variation in benthic invertebrate fatty acid profiles (Fig. 2) suggests that the quality of the benthic invertebrates' diets may have changed over the course of the experiment, leading to less EPA and ALA, and greater LIN and ARA content in benthic invertebrates in the latter weeks of the experiment. Our experiment was conducted in the spring (April–May), so this change may have been due to a pulse of terrestrial leaf litter, particulate and dissolved carbon, and subsequent periphyton growth associated with spring melting and warming. Thus, these differences in dietary fatty acids consumed by stream invertebrates suggest a shift from autochthonous to allochthonous carbon sources. This explanation is also supported by the somewhat large random effect of time we observed in our mixed‐effects ANOVA models for LIN (marginal *R* ^2^, fixed factors only, $R_{m}^{2}$ = 0.30; conditional *R* ^2^, fixed and random factors, $R_{c}^{2}$ = 0.53) and ARA ($R_{m}^{2}$ = 0.44; $R_{c}^{2}$ = 0.83), which are more abundant in terrestrial food sources than aquatic food sources (Appendix S1: Table S4). Importantly, however, this variability of fatty acid content in benthic invertebrates did not affect their fatty acid stable isotope values, which had lower variance than the fish pellets (Fig. 4). Although there were some differences in fatty acid content between earthworms and benthic invertebrates, these differences were not well reflected in the fatty acid profiles of the respective treatments in fish muscle and liver tissues, suggesting that the retention of fatty acids in fish did not directly reflect its dietary fatty acid supply. This result is similar to previous studies with other aquatic consumers (Bec et al. 2003, Brett et al. 2009, Heissenberger et al. 2010), and calls for more precise diet‐tracing methods. However, similar to bulk δ^13^C, the differences in δ^13^C of dietary PUFA were also not well reflected by the δ^13^C_PUFA_ in fish liver and muscle (Fig. 4). While diets had significantly different δ^13^C for some PUFA, these same differences were not seen in the respective treatment groups in fish muscle and liver.

Contrary to our expectations, we observed a significant difference in δ^13^C between benthic and terrestrial invertebrates; δ^13^C values of earthworms were more enriched relative to benthic invertebrates. The differences in fish muscle or liver were not significant, but livers from fish with a terrestrial diet were more enriched in δ^13^C relative to fish with a benthic diet, and fish with a mixed diet had intermediate δ^13^C values (Fig. 3d). This pattern indicates that if more time had allowed for greater tissue turnover, using bulk isotopes may in fact have been sufficient for partitioning these selected diet items. This isotopic difference may be due to the use of earthworms as the terrestrial diet treatment. Because earthworms are detritivores and feed on potentially dozens of materials (Bernier 1998), they likely have unique δ^13^C values relative to the terrestrial invertebrates that more commonly subsidize fish diets in temperate and montane lakes, such as arthropods, and winged insects (Mehner et al. 2005, Vander Zanden and Gratton 2011).

The δ^13^C_PUFA_ values in benthic invertebrates and earthworms had low variability, with the exception of δ^13^C_DHA_ (Fig. 4). In fact, the variability of δ^13^C_PUFA_ in the treatment diets was often less than in the baseline diet (i.e., pellets). This result is encouraging for field studies because it suggests that δ^13^C_PUFA_ in invertebrates from a given system is relatively consistent for most PUFA, even when coarse functional groups are used. Although the differences in δ^13^C_PUFA_ among treatments in fish tissues were not significant, the patterns of δ^13^C_ALA_ we observed in fish liver and the patterns of δ^13^C_EPA_ in fish liver and muscle suggest that a longer experimental period that allows for greater tissue turnover may yield more pronounced results. The trophic discrimination factors (Δ^13^C_FA_) we calculated from fish with the baseline diet support this hypothesis. The δ^13^C values of LIN, ALA, and EPA in fish tissues were similar to the δ^13^C in the pellets (Δ^13^C = 0.3–2.7‰), indicating that when fish have sufficient dietary supply of these PUFA, the δ^13^C_PUFA_ in its tissues is similar to that of its diet, but somewhat enriched due to metabolic processes (Caut et al. 2009). Because tissue turnover is faster in fish liver than in fish muscle, we suggest that future diet mixing studies prioritize measuring δ^13^C_FA_ in the liver, and secondarily, muscle tissue (Heady and Moore 2013, Mohan et al. 2016).

The use of consumer–diet ratios worked well for identifying which PUFA could be reliable dietary tracers. For example, the high consumer–diet ratio for LIN in fish with terrestrial diets (Table 2) may explain why δ^13^C_LIN_ was significantly more depleted in fish liver from the terrestrial diet treatment compared with fish from mixed and baseline diets (Fig. 4). Fish feeding on terrestrial invertebrates were likely not getting enough dietary LIN, and therefore allocated LIN from their lipid stores to meet their physiological LIN demand, leading to a relatively depleted δ^13^C_LIN_ value. Mobilizing LIN from lipid stores would mean that the δ^13^C_LIN_ value would be representative of the repository LIN (i.e., δ^13^C_LIN_ in pellets), as opposed to dietary LIN, and could thus explain why differences were seen in fish with terrestrial diets, even though there were no differences in δ^13^C_LIN_ between diet treatments.

Docosahexaenoic acid had a high consumer–diet ratio and, as expected, was not a reliable PUFA for tracing δ^13^C. In fact, likely because of the very low DHA content in benthic invertebrates, the δ^13^C_DHA_ values we obtained were not reliable. Because dietary DHA was so low in both benthic invertebrates and earthworms (Appendix S1: Table S3), fish were likely converting repository DHA to meet their physiological DHA demand, which is why the δ^13^C_DHA_ in fish tissues was similar, or somewhat depleted relative to pellet δ^13^C_DHA_ (Heissenberger et al. 2010, Murray et al. 2014). Other studies have observed a δ^13^C depletion of 1–4‰ in fatty acids of a consumer relative to its diet (Budge et al. 2011), and the depletion in muscle and liver δ^13^C_DHA_ relative to pellet δ^13^C_DHA_ falls within this range (Fig. 4).

The δ^13^C_ARA_ values in fish from all treatments were enriched relative to benthic invertebrates and pellets, but similar to the δ^13^C_ARA_ values in earthworms. We suspect this is because ARA content in pellets was low (Appendix S1: Table S3), and thus an insufficient dietary supply for fish (Table 2). Conversely, ARA was abundant in earthworms (Appendix S1: Table S3), and so the δ^13^C_ARA_ in fish with the terrestrial diet was similar to the δ^13^C_ARA_ of earthworms (Fig. 4, Table 3).

We hypothesize that the general lack of significant differences in δ^13^C values between treatments in fish tissues is due to variability introduced by low sample size, constraints in the number of treatment tanks, and diet competition among fish, as well as the duration of the experiment. Only 2/10 fish from the benthic treatment, 6/10 fish from the terrestrial treatment, and 10/20 fish from the mixed treatment gained weight during the experiment, which we attribute to diet competition among fish. Such competition was likely in part due to the challenge of collecting enough biomass of each of the diets in a single day, and our inability to keep fish in separate tanks (we attempted previous experiments this way, but the fish did not feed when alone). As such, we would expect the differences between fish tissue treatments would be much more pronounced with a larger sample size, longer experiment time, and more substantial fish weight gain in future experiments. A comparison of the fatty acid content and δ^13^C between fish that gained weight and lost weight during the experiment supports this conclusion. We used a Welch two‐sample *t* test with a Hommel correction to determine whether there were differences in fatty acid content and δ^13^C between fish with weight gain and weight loss for each diet treatment. Fish with positive weight gain had significantly higher liver ALA content in benthic (*t* = −5.69 df = 6, *P* < 0.01) and mixed (*t* = −2.80, df = 14, *P* = 0.03) treatments, as well as more depleted δ^13^C_ALA_ (*t* = 5.49, df = 5, *P* < 0.01) and δ^13^C_DHA_ (*t* = 4.09, df = 6, *P* = 0.02) values in terrestrial treatments relative to fish that lost weight (Appendix S1: Figs. S1, S2). Additionally, there appeared to be moderate non‐significant differences in means between other groups with lower sample sizes (e.g., δ^13^C_DHA_ in muscle and liver of fish with benthic diets, Appendix S1: Fig. S2). These differences support our assumption that competition led to insufficient dietary supply of certain fatty acids for fish that lost weight, and reinforces our decision to use only fish with weight gain in our analyses.

Our results suggest that understanding the physiological context of a system—including dietary availability of PUFA, consumer–diet ratios, and allocation of pre‐formed PUFA from fish storage lipids—is crucial to successfully using fatty acid stable isotopes in diet‐tracing studies. Similar studies have led others to conclude that fatty acid stable isotopes could be used in the field conservatively, but clearly more laboratory studies are needed to decipher how consumers fractionate dietary PUFA and other fatty acids (Budge et al. 2008, 2011, 2016, Bec et al. 2011). We simulated field conditions by using live prey and allowing for competition and resource‐limitation, and we feel the complexity of our results warrants significant trepidation with field applications. However, we do identify a number of suggestions to determine the suitability of fatty acid stable isotopes for a given system. Our use of consumer–diet ratios illustrates the importance of dietary availability of PUFA when choosing tracers, but future work could also incorporate analysis of mead acid, which can be an indicator of essential fatty acid deficiency (Holman 1960, Ichi et al. 2014). Further, DHA is rarely available to consumers in terrestrial and benthic freshwater prey (Tocher 2003), so we suggest excluding this PUFA as a possible δ^13^C_FA_ candidate for diet tracing in freshwater fishes. Our study simulates the trends that might be expected in oligotrophic, biomass‐limited, and dietary DHA‐limited freshwater systems such as northern temperate, arctic, or alpine waterbodies. Therefore, similar patterns of fractionation and variability with EPA and DHA may be possible in these systems. However, warmer and more productive systems may be subject to fewer confounding issues of resource availability and lipid metabolism. Therefore, we encourage future work to investigate the effect of temperature on fractionation processes and the utility of fatty acid stable isotopes in aquatic consumers.

This is the first study to examine how stable carbon isotopes of fatty acids can partition between dietary autochthonous and allochthonous energy sources in freshwater consumers. Our results indicate that although stable carbon isotopes of fatty acids may overcome some of the challenges of bulk stable isotopes, this method comes with its own complex set of challenges and assumptions. The differences we observed in δ^13^C values of ALA, ARA, and EPA in diet items, along with the low trophic discrimination factors for baseline fish feeding on pellets, suggest that stable carbon isotopes of fatty acids could be effective under certain conditions. If the supply of these fatty acids is high enough in diets to meet the consumer's physiological demand, fatty acid δ^13^C could increase the number of sources in, and therefore the reliability and accuracy of isotope mixing models. However, such a perfect scenario is unlikely in the field, and thus, the effectiveness of PUFA δ^13^C values as biomarkers in consumers will depend heavily on ecosystem productivity (i.e., consumer food availability), physiological context, and sample size. As such, we recommend against the use of this as a primary diet‐tracing method in field studies until more laboratory studies have been conducted. Although more work is needed to learn about the nuances of fatty acid stable isotope biomarkers across different systems, our findings provide important baseline knowledge for future studies using fatty acid stable isotopes to trace carbon energy flow in aquatic food webs.
