# Supplementary material for: Fatty acid stable isotopes add clarity, but also complexity, to tracing energy pathways in aquatic food webs
Source: Ecosphere. 2021 Feb 7;12(2):e03360. doi: 10.1002/ecs2.3360 (PMC8641385; doi:10.1002/ecs2.3360)
Supplement: Supplementary file 1 [file ECS2-12-e03360-s001.pdf]

Ecosphere - Fatty acid stable isotopes add clarity, but also complexity, to tracing energy pathways in aquatic food webs

Chiapella, A., Kainz, M., Strecker, A.

Appendix S1

Table S1. Ingredients for Aqua Uni 4mm pellets.

Ingredient	Percent composition
Poultry meal	18.5
Sunflower concentrate	18.0
Fish meal	14.0
Wheat	13.7
Swine blood meal	8.8
Wheat meal	7.7
Rapeseed cake	7.5
Fish oil	5.9
Rapeseed oil	5.3
Added minerals	0.6

Table S2. Stream benthic invertebrates collected for benthic treatment diets.

**Family**	**Taxa**	**Feeding strategy**
Ephemeroptera	*Ecdyonurus venosus*	Scrapers, collectors
	Ecdyonurus forcipula	Scrapers, collectors
Plecoptera	*Perlodes microcephala*	Predators
	Isoperla similis	Predators
Trichoptera	*Sericostoma* sp.	Shredders
	Drusus trifidus	Shredders
	Potamophylax rotundipennis	Shredders, scrapers
Oligochaeta	*Lumbricidae*	Decomposers

Ecosphere - Fatty acid stable isotopes add clarity, but also complexity, to tracing energy pathways in aquatic food webs
Chiapella, A., Kainz, M., Strecker, A.

Table S3. Average concentrations (± standard deviation) of each major fatty acid group and individual PUFA in diets & treatments.

	SAFA (mg/g)	MUFA (mg/g)	PUFA (mg/g)	ALA (mg/g)	ARA (mg/g)	LIN (mg/g)	EPA (mg/g)	DHA (mg/g)
Earthworms	18.32 ± 4.41	16.52 ± 7.38	61.01 ± 4.21	2.28 ± 3.33	8.79 ± 13.53	3.99 ± 5.77	28.65 ± 42.85	0.22 ± 0.10
Benthic Invertebrates	27.70 ± 2.73	29.49 ± 4.15	40.92 ± 2.51	127.05 ± 130.72	5.70 ± 5.87	35.00 ± 34.26	96.69 ± 101.41	1.22 ± 0.77
Pellets	17.65 ± 0.88	46.33 ± 1.47	35.93 ± 0.63	5.09 ± 0.43	0.42 ± 0.05	18.36 ±1.94	3.40 ± 0.83	2.53 ± 1.91
Fish liver (benthic)	17.89 ± 7.01	46.92 ± 16.97	34.55 ± 11.07	4.87 ± 3.19	4.71 ± 0.75	20.58 ± 8.50	8.11 ± 2.95	19.95 ± 5.91
Fish liver (control)	14.41 ± 1.62	64.28 ± 3.06	21.18 ± 2.35	4.13 ± 0.82	4.01 ± 0.50	22.35 ± 4.64	6.54 ± 1.03	16.46 ± 2.42
Fish liver (mixed)	17.74 ± 5.11	46.98 ± 14.86	35.08 ± 10.74	7.60 ± 9.48	6.35 ± 4.12	41.15 ± 58.84	10.55 ± 7.30	25.68 ± 16.98
Fish liver (terrestrial)	16.81 ± 4.89	44.73 ± 14.56	38.17 ± 11.04	2.78 ± 1.39	5.03 ± 1.03	15.37 ± 6.14	6.62 ± 2.01	19.99 ± 3.93
Fish muscle (benthic)	19.98 ± 4.23	42.15 ± 9.36	37.72 ± 5.32	3.27 ± 1.95	1.36 ± 0.50	15.74 ± 11.06	4.24 ±1.25	11.72 ± 2.83
Fish muscle (control)	17.67 ± 0.48	51.43 ± 1.51	30.78 ± 1.64	6.91 ± 0.95	1.54 ± 0.30	29.45 ± 4.87	5.73 ± 1.30	13.96 ± 3.07
Fish muscle (mixed)	19.83 ± 3.88	42.77 ± 11.51	37.21 ± 7.74	4.62 ± 2.52	1.41 ± 0.37	20.81 ± 11.25	5.12 ± 2.19	14.08 ± 4.37
Fish muscle (terrestrial)	20.61 ± 4.80	40.88 ± 13.05	38.33 ± 8.39	3.50 ± 1.44	1.28 ± 0.32	15.89 ± 6.04	4.08 ± 1.31	11.72 ± 2.98

Table S4. Full model results for mixed-effects ANOVAs and Tukey's adjusted LSM differences of individual PUFA concentrations between treatment groups for (a) diets, (b) fish muscle, and (c) fish liver (for fish with weight gain only). Sum Sq = sum of squares; mean sq= mean square; SE= standard error; DF= degrees of freedom.

(a) Diets

[ALA]

ANOVA:	Sum Sq	Mean Sq	NumDF	DenDF	F	p
	59.49	29.74	2	12.99	3.77	0.05

Random effect:	Variance	SD
time	0.00	0.00
residual	1.68	1.30

LSM differences

treatment	ß	SE	DF	*t*-value	Lower CI	Upper CI	p
benthic - baseline	122.00	49.40	13	2.47	15.21	229.00	0.03
benthic - terrestrial	124.80	61.30	13	2.04	-7.65	257.00	0.06
baseline - terrestrial	2.80	62.80	13	0.04	-132.88	138.00	0.97

[LIN]

ANOVA:	Sum Sq	Mean Sq	NumDF	DenDF	F	p
	321.00	160.50	2	10.72	3.46	0.07

Random effect:	Variance	SD
time	212.40	14.58
residual	463.30	21.52

LSM differences

treatment	ß	SE	DF	*t*-value	Lower CI	Upper CI	p
benthic - baseline	33.00	16.05	8.60	2.05	-3.63	69.60	0.07
benthic - terrestrial	34.60	15.04	10.90	2.30	1.50	67.80	0.04
baseline - terrestrial	1.70	17.34	12.80	0.10	-35.87	39.20	0.93

[ARA]

ANOVA:	Sum Sq	Mean Sq	NumDF	DenDF	F	p	
	419.90	209.95	2	11.90	8.72	0.01	
Random effect:	Variance	SD					
time	54.84	7.41					
residual	24.07	4.91					
	LSM differences						
treatment	ß	SE	DF	*t*-value	Lower CI	Upper CI	p
benthic - baseline	15.50	4.15	12.7	3.75	6.55	24.50	0.00
benthic - terrestrial	-0.80	3.46	11.0	-0.24	-8.42	6.79	0.82
baseline - terrestrial	-16.30	4.23	12.1	-3.86	-25.56	-7.13	0.00

[EPA]

ANOVA:	Sum Sq	Mean Sq	NumDF	DenDF	F	p	
	296.42	148.21	2	13.00	2.95	0.09	
Random effect:	Variance	SD					
time	0.00	0.00					
residual	0.01	0.70					
	LSM differences						
treatment	ß	SE	DF	*t*-value	Lower CI	Upper CI	p
benthic - baseline	93.30	39.50	13	2.36	8.06	178.50	0.03
benthic - terrestrial	68.00	48.90	13	1.39	-37.68	173.80	0.19
baseline - terrestrial	-25.30	50.10	13	-0.50	-133.59	83.1	0.62

[DHA]

ANOVA:	Sum Sq	Mean Sq	NumDF	DenDF	F	p	
treatment	11.87	5.93	2	13	3.54	0.061	
Random effect:	Variance	SD					
time	0.00	0.00					
residual	1.68	1.30					
	LSM differences						
treatment	ß	SE	DF	*t*-value	Lower CI	Upper CI	p
benthic - baseline	-1.30	0.72	13	-1.82	-2.87	0.25	0.09
benthic - terrestrial	1.00	0.89	13	1.13	-0.92	2.94	0.28
baseline - terrestrial	2.30	0.92	13	2.53	0.34	4.30	0.03

(b) Muscle

[ALA]

ANOVA:	Sum Sq	Mean Sq	NumDF	DenDF	F	p
	20.28	6.76	3	4	2.13	0.23

Random effect:	Variance	SD
tank	0.90	0.95
residual	3.18	1.78

treatment	ß	SE	DF	*t*-value	Lower CI	Upper CI	p
	LSM Differences						
benthic - baseline	-4.40	1.89	5.4	-2.33	-9.17	0.36	0.06
benthic - mixed	-2.30	1.61	10.1	-1.44	-5.90	1.27	0.18
benthic - terrestrial	-1.30	1.74	8.2	-0.74	-5.27	2.70	0.48
baseline - mixed	2.10	1.45	2.6	1.45	-2.99	7.18	0.26
baseline - terrestrial	3.10	1.59	2.6	1.97	-2.38	8.63	0.16
mixed - terrestrial	1.00	1.24	3.9	0.83	-2.46	4.52	0.45

[LIN]

ANOVA:	Sum Sq	Mean Sq	NumDF	DenDF	F	p
	623.61	207.87	3	7	2.91	0.11

Random effect:	Variance	SD
tank	3.10	1.76
residual	71.38	8.45

treatment	ß	SE	DF	*t*-value	Lower CI	Upper CI	p
	LSM Differences						
benthic - baseline	-19.40	7.39	5.0	-2.62	-38.40	-0.39	0.05
benthic - mixed	-11.30	6.73	11.5	-1.69	-26.10	3.38	0.12
benthic - terrestrial	-6.50	7.12	9.3	-0.92	-22.60	9.48	0.38
baseline - mixed	8.00	5.04	1.4	1.60	-26.10	42.16	0.30
baseline - terrestrial	12.80	5.55	1.5	2.31	-21.20	46.88	0.19
mixed - terrestrial	4.80	4.63	2.8	1.03	-10.40	20.03	0.38

[ARA]

ANOVA:	Sum Sq	Mean Sq	NumDF	DenDF	F	p
	0.34	0.11	3	6	0.83	0.52

Random effect:	Variance	SD
tank	0.00	0.02
residual	0.13	0.37

	LSM Differences						
treatment	ß	SE	DF	*t*-value	Lower CI	Upper CI	p
benthic - baseline	-0.40	0.31	5.0	-1.34	-1.21	0.38	0.20
benthic - mixed	-0.40	0.29	12.0	-1.25	-0.98	0.26	0.20
benthic - terrestrial	-0.20	0.30	9.7	-0.65	-0.87	0.48	0.50
baseline - mixed	0.10	0.20	1.2	0.28	-1.81	1.92	0.80
baseline - terrestrial	0.20	0.22	1.3	0.97	-1.53	1.96	0.50
mixed - terrestrial	0.20	0.19	2.6	0.84	-0.50	0.82	0.50

[EPA]

ANOVA:	Sum Sq	Mean Sq	NumDF	DenDF	F	p
	6.24	2.08	3	3	0.77	0.57

Random effect:	Variance	SD
tank	0.28	0.53
residual	2.70	1.64

	LSM Differences						
treatment	ß	SE	DF	*t*-value	Lower CI	Upper CI	p
benthic - baseline	-1.80	1.52	5.5	-1.18	-5.59	2.01	0.30
benthic - mixed	-1.40	1.36	11.5	-1.04	-4.38	1.55	0.30
benthic - terrestrial	-0.50	1.44	9.4	-0.34	-3.73	2.76	0.70
baseline - mixed	0.40	1.08	1.9	0.35	-4.52	5.27	0.80
baseline - terrestrial	1.30	1.19	2.0	1.10	-3.84	6.45	0.40
mixed - terrestrial	0.90	0.97	3.4	0.96	-1.94	3.79	0.40

[DHA]							
ANOVA:	Sum Sq	Mean Sq	NumDF	DenDF	F	p	
	32.16	10.72	3	19	0.98	0.42	
Random effect:	Variance	SD					
tank	0.00	0.00					
residual	0.11	3.30					
	LSM Differences						
treatment	ß	SE	DF	*t*-value	Lower CI	Upper CI	p
benthic - baseline	-3.00	2.76	19	-1.08	-8.76	2.81	0.30
benthic - mixed	-3.70	2.56	19	-1.43	-9.01	1.69	0.20
benthic - terrestrial	-1.50	2.70	19	-0.57	-7.18	4.10	0.60
baseline - mixed	-0.70	1.81	19	-0.38	-4.47	3.10	0.70
baseline - terrestrial	1.40	2.00	19	0.72	-2.75	5.62	0.50
mixed - terrestrial	2.10	1.71	19	1.24	-1.45	5.69	0.20

(c) Liver

[ALA]

ANOVA:	Sum Sq	Mean Sq	NumDF	DenDF	F	p
	19.14	6.38	3.00	3.61	3.11	0.16
Random effect:	Variance	SD				
tank	1.77	1.33				
residual	2.05	1.43				

	LSM Differences						
treatment	ß	SE	DF	*t*-value	Lower CI	Upper CI	p
benthic - baseline	4.00	2.02	3.90	1.99	-1.68	9.72	0.12
benthic - mixed	3.70	1.61	6.20	2.28	-0.24	7.56	0.06
benthic - terrestrial	5.30	1.77	5.20	3.02	0.84	9.85	0.03
baseline - mixed	-0.40	1.69	2.60	-0.21	-6.24	5.52	0.85
baseline - terrestrial	1.30	1.85	2.60	0.72	-5.11	7.76	0.53
mixed - terrestrial	1.70	1.38	3.20	1.23	-2.52	5.89	0.30

[LIN]

ANOVA:	Sum Sq	Mean Sq	NumDF	DenDF	F	p
	264.51	88.17	3.00	5.46	2.96	0.13
Random effect:	Variance	SD				
tank	17.38	4.17				
residual	29.82	5.46				

	LSM Differences						
treatment	ß	SE	DF	*t*-value	Lower CI	Upper CI	p
benthic - baseline	8.20	6.85	6.00	1.20	-8.57	25.00	0.28
benthic - mixed	12.00	5.58	9.60	2.15	-0.51	24.50	0.06
benthic - terrestrial	17.70	6.10	8.10	2.90	3.66	31.70	0.02
baseline - mixed	3.80	5.55	3.80	0.68	-12.04	19.60	0.53
baseline - terrestrial	9.50	6.08	3.80	1.56	-7.81	26.80	0.20
mixed - terrestrial	5.70	4.60	4.90	1.24	-6.22	17.60	0.27

[ARA]							
ANOVA:	Sum Sq	Mean Sq	NumDF	DenDF	F	p	
	3.99	1.33	3.00	4.46	1.32	0.38	
Random effect:	Variance	SD					
tank	0.08	0.29					
residual	1.01	1.01					
	LSM Differences						
treatment	ß	SE	DF	*t*-value	Lower CI	Upper CI	p
benthic - baseline	1.50	0.91	6.90	1.60	-0.70	3.62	0.20
benthic - mixed	0.40	0.82	13.20	0.47	-1.38	2.15	0.60
benthic - terrestrial	0.30	0.87	11.20	0.35	-1.60	2.22	0.70
baseline - mixed	-1.10	0.64	2.40	-1.68	-3.42	1.26	0.20
baseline - terrestrial	-1.20	0.70	2.50	-1.64	-3.64	1.33	0.20
mixed - terrestrial	-0.10	0.58	4.40	-0.13	-1.62	1.47	0.90

[EPA]							
ANOVA:	Sum Sq	Mean Sq	NumDF	DenDF	F	p	
	30.55	10.18	3.00	19.00	1.87	0.17	
Random effect:	Variance	SD					
tank	0.00	0.00					
residual	5.45	2.33					
	LSM Differences						
treatment	ß	SE	DF	*t*-value	Lower CI	Upper CI	p
benthic - baseline	3.90	1.95	19.00	2.00	-0.19	7.98	0.06
benthic - mixed	2.10	1.81	19.00	1.15	-1.71	5.86	0.27
benthic - terrestrial	3.60	1.91	19.00	1.89	-0.38	7.59	0.07
baseline - mixed	-1.80	1.28	19.00	-1.42	-4.50	0.85	0.17
baseline - terrestrial	-0.30	1.41	19.00	-0.21	-3.25	2.67	0.84
mixed - terrestrial	1.50	1.21	19.00	1.27	-0.99	4.05	0.22

[DHA]							
ANOVA:	Sum Sq	Mean Sq	NumDF	DenDF	F	p	
	50.63	16.88	3.00	3.80	0.94	0.50	
Random effect:	Variance	SD					
tank	1.85	1.36					
residual	17.87	4.23					
	LSM Differences						
treatment	ß	SE	DF	*t*-value	Lower CI	Upper CI	p
benthic - baseline	2.90	3.91	5.90	0.74	-6.70	12.50	0.50
benthic - mixed	-0.10	3.49	12.00	-0.03	-7.71	7.48	1.00
benthic - terrestrial	-2.20	3.71	9.90	-0.60	-10.52	6.04	0.60
baseline - mixed	-3.00	2.78	2.10	-1.08	-14.54	8.52	0.40
baseline - terrestrial	-5.10	3.06	2.20	-1.68	-17.33	7.05	0.20
mixed - terrestrial	-2.10	2.49	3.70	-0.86	-9.23	4.97	0.40

Table S5. Full model results for mixed-effects ANOVAs and Tukey's adjusted LSM differences of individual PUFA and bulk $\delta^{13}C$ between treatment groups for (a) diets, (b) fish muscle, and (c) fish liver (for fish with weight gain only).

(a) Diets

$\delta^{13}C_{ALA}$

ANOVA:	Sum Sq	Mean Sq	NumDF	DenDF	F	p	
	63.26	31.63	2	10	10.66	0.003	
Random effect:	Variance	SD					
time	0.00	0.00					
residual	2.97	1.72					
	LSM differences						
treatment	ß	SE	DF	*t*-value	Lower CI	Upper CI	p
benthic - baseline	-2.80	1.19	10	-2.35	-5.44	-0.14	0.04
benthic - terrestrial	-5.30	1.19	10	-4.50	-7.99	-2.70	0.00
baseline - terrestrial	-2.60	1.41	10	-1.82	-5.69	0.58	0.10

$\delta^{13}C_{LIN}$

ANOVA:	Sum Sq	Mean Sq	NumDF	DenDF	F	p	
	1.06	0.53	2	10	0.32	0.73	
Random effect:	Variance	SD					
time	0.00	0.00					
residual	1.64	1.28					
	LSM differences						
treatment	ß	SE	DF	*t*-value	Lower CI	Upper CI	p
benthic - baseline	-0.70	0.88	10	-0.77	-2.65	1.29	0.50
benthic - terrestrial	-0.40	0.88	10	-0.44	-2.36	1.58	0.70
baseline - terrestrial	0.30	1.05	10	0.28	-2.04	2.62	0.80

$\delta^{13}C_{ARA}$

ANOVA:	Sum Sq	Mean Sq	NumDF	DenDF	F	p
	79.80	39.9	2	10	19.74	0.00
Random effect:	Variance	SD				
time	0.00	0.00				
residual	2.02	1.42				

LSM differences

treatment	ß	SE	DF	*t*-value	Lower CI	Upper CI	p
benthic - baseline	-1.20	0.98	10	-1.27	-3.44	0.94	0.23
benthic - terrestrial	-6.10	0.98	10	-6.25	-8.32	-3.95	0.00
baseline - terrestrial	-4.90	1.16	10	-4.21	-7.47	-2.30	0.00

$\delta^{13}C_{EPA}$

ANOVA:	Sum Sq	Mean Sq	NumDF	DenDF	F	p
	63.83	31.92	2	8.4448	22.02	0.00
Random effect:	Variance	SD				
time	0.18	0.42				
residual	1.45	1.20				

LSM differences

treatment	ß	SE	DF	*t*-value	Lower CI	Upper CI	p
benthic - baseline	-5.90	0.92	6.5	-6.38	-8.064	-3.66	0.00
benthic - terrestrial	-3.10	0.84	8.7	-3.73	-5.015	-1.21	0.01
baseline - terrestrial	2.70	1.03	9.9	2.67	0.448	5.04	0.02

$\delta^{13}C_{DHA}$

ANOVA:	Sum Sq	Mean Sq	NumDF	DenDF	F	p
	22.20	22.20	1	2.01	110.32	0.01
Random effect:	Variance	SD				
time	149.37	12.22				
residual	0.20	0.45				

LSM differences

treatment	ß	SE	DF	*t*-value	Lower CI	Upper CI	p
baseline - terrestrial	5.40	0.52	2	10.50	3.23	7.65	0.01

Ecosphere - Fatty acid stable isotopes add clarity, but also complexity, to tracing energy pathways in aquatic food webs

Chiapella, A., Kainz, M., Strecker, A.

	Bulk $\delta^{13}C$						
ANOVA:	Sum Sq	Mean Sq	NumDF	DenDF	F	p	
	104.66	52.33	2.00	6.00	9.87	0.01	
Random effect:	Variance	SD					
time	0.00	0.00					
residual	5.30	2.30					
	LSM differences						
treatment	ß	SE	DF	*t*-value	Lower CI	Upper CI	p
benthic - baseline	-7.60	1.76	6	-4.30	-11.87	-3.26	0.01
benthic - terrestrial	-5.30	1.99	6	-2.65	-10.18	-0.41	0.04
baseline - terrestrial	2.30	2.10	6	1.08	-2.87	7.42	0.32

(b) Muscle

$\delta^{13}C_{ALA}$

ANOVA:	Sum Sq	Mean Sq	NumDF	DenDF	F	p	
	0.07	0.02	3.00	4.32	0.01	1.00	
Random effect:	Variance	SD					
tank	13.16	3.63					
residual	1.88	1.37					
	LSM differences						
treatments	ß	SE	DF	*t*-value	Lower CI	Upper CI	p
benthic - baseline	0.40	4.59	4	0.08	-12.01	12.75	0.90
benthic - mixed	-0.20	3.32	5	-0.07	-8.90	8.45	0.90
benthic - terrestrial	-0.50	3.80	5	-0.12	-10.51	9.59	0.90
baseline - mixed	-0.60	4.13	4	-0.15	-12.00	10.80	0.90
baseline - terrestrial	-0.80	4.52	4	-0.18	-13.33	11.65	0.90
mixed - terrestrial	-0.20	3.22	4	-0.07	-9.02	8.55	0.90

$\delta^{13}C_{LIN}$

ANOVA:	Sum Sq	Mean Sq	NumDF	DenDF	F	p	
	0.38	0.13	3.00	19.00	0.21	0.89	
Random effect:	Variance	SD					
tank	0.00	0.00					
residual	0.60	0.78					
	LSM differences						
	ß	SE	DF	*t*-value	Lower CI	Upper CI	p
benthic - baseline	-0.50	0.65	19	-0.72	-1.83	0.89	0.50
benthic - mixed	-0.50	0.60	19	-0.77	-1.72	0.80	0.50
benthic - terrestrial	-0.40	0.63	19	-0.71	-1.77	0.88	0.50
baseline - mixed	0.00	0.42	19	0.01	-0.88	0.90	1.00
baseline - terrestrial	0.00	0.47	19	0.04	-0.97	1.00	1.00
mixed - terrestrial	0.00	0.40	19	0.03	-0.83	0.85	1.00

$\delta^{13}C_{ARA}$

ANOVA:	Sum Sq	Mean Sq	NumDF	DenDF	F	p	
	28.10	9.37	3.00	19.00	2.95	0.06	
Random effect:	Variance	SD					
tank	0.00	0.00					
residual	3.18	1.78					
	LSM differences						
treatment	ß	SE	DF	*t*-value	Lower CI	Upper CI	p
benthic - baseline	4.30	1.49	19	2.87	1.16	7.40	0.01
benthic - mixed	3.00	1.38	19	2.16	0.09	5.87	0.04
benthic - terrestrial	2.40	1.46	19	1.62	-0.69	5.40	0.12
baseline - mixed	-1.30	0.98	19	-1.33	-3.35	0.74	0.20
baseline - terrestrial	-1.90	1.08	19	-1.78	-4.18	0.34	0.09
mixed - terrestrial	-0.60	0.92	19	-0.67	-2.55	1.31	0.51

$\delta^{13}C_{EPA}$

ANOVA:	Sum Sq	Mean Sq	NumDF	DenDF	F	p	
	5.93	1.98	3.00	3.84	0.67	0.61	
Random effect:	Variance	SD					
tank	0.77	0.88					
residual	2.94	1.71					
	LSM differences						
treatment	ß	SE	DF	*t*-value	Lower CI	Upper CI	p
benthic - baseline	1.30	1.79	5	0.75	-3.30	5.99	0.50
benthic - mixed	1.50	1.53	10	0.99	-1.92	4.96	0.30
benthic - terrestrial	0.10	1.65	8	0.08	-3.70	3.96	0.90
baseline - mixed	0.20	1.36	2	0.13	-5.16	5.51	0.90
baseline - terrestrial	-1.20	1.49	2	-0.81	-6.97	4.54	0.50
mixed - terrestrial	-1.40	1.17	3	-1.19	-4.86	2.08	0.30

$\delta^{13}C_{DHA}$

ANOVA:	Sum Sq	Mean Sq	NumDF	DenDF	F	p
	14.73	4.91	3.00	4.81	1.26	0.39
Random effect:	Variance	SD				
tank	105.42	10.27				
residual	3.91	1.98				

	LSM differences						
treatment	b	SE	DF	t	Lower CI	Upper CI	p
benthic - baseline	-15.30	12.68	5	-1.21	-48.30	17.68	0.30
benthic - mixed	-16.30	9.03	5	-1.81	-39.60	6.98	0.10
benthic - terrestrial	-16.50	10.39	5	-1.59	-43.40	10.44	0.20
baseline - mixed	-1.00	11.53	5	-0.09	-31.20	29.16	0.90
baseline - terrestrial	-1.20	12.63	5	-0.09	-34.20	31.88	0.90
mixed - terrestrial	-0.20	8.95	5	-0.02	-23.50	23.18	1.00

Bulk $\delta^{13}C$

ANOVA:	Sum Sq	Mean Sq	NumDF	DenDF	F	p
	4.71	1.57	3.00	16.00	2.53	0.09
Random effect:	Variance	SD				
tank	0.00	0.00				
residual	0.62	0.79				

	LSM differences						
treatment	ß	SE	DF	*t*-value	Lower CI	Upper CI	p
benthic - baseline	1.80	0.79	16.00	2.35	0.18	3.52	0.03
benthic - mixed	0.90	0.61	16.00	1.42	-0.43	2.16	0.17
benthic - terrestrial	0.30	0.64	16.00	0.52	-1.03	1.70	0.61
baseline - mixed	-1.00	0.61	16.00	-1.61	-2.28	0.31	0.13
baseline - terrestrial	-1.50	0.64	16.00	-2.36	-2.88	-0.16	0.03
mixed - terrestrial	-0.50	0.41	16.00	-1.32	-1.40	0.33	0.21

(c) Liver

$\delta^{13}C_{ALA}$

ANOVA:	Sum Sq	Mean Sq	NumDF	DenDF	F	p	
	52.24	17.41	3.00	19.00	1.12	0.37	
Random effect:	Variance	SD					
tank	0.00	0.00					
residual	15.54	3.94					
	LSM differences						
treatment	ß	SE	DF	*t*-value	Lower CI	Upper CI	p
benthic - baseline	5.70	3.30	19	1.74	-1.15	12.65	0.10
benthic - mixed	4.80	3.05	19	1.59	-1.55	11.23	0.10
benthic - terrestrial	3.70	3.22	19	1.16	-2.99	10.48	0.30
baseline - mixed	-0.90	2.16	19	-0.42	-5.42	3.61	0.70
baseline - terrestrial	-2.00	2.39	19	-0.84	-7.00	2.99	0.40
mixed - terrestrial	-1.10	2.04	19	-0.54	-5.36	3.16	0.60

$\delta^{13}C_{LIN}$

ANOVA:	Sum Sq	Mean Sq	NumDF	DenDF	F	p	
	12.55	4.18	3.00	19.00	5.01	0.01	
Random effect:	Variance	SD					
tank	0.00	0.00					
residual	0.84	0.91					
	LSM differences						
treatment	ß	SE	DF	*t*-value	Lower CI	Upper CI	p
benthic - baseline	-0.90	0.77	19	-1.13	-2.46	0.74	0.27
benthic - mixed	0.30	0.71	19	0.37	-1.22	1.74	0.72
benthic - terrestrial	1.30	0.75	19	1.70	-0.29	2.83	0.11
baseline - mixed	1.10	0.50	19	2.25	0.08	2.17	0.04
baseline - terrestrial	2.10	0.55	19	3.85	0.97	3.29	0.00
mixed - terrestrial	1.00	0.47	19	2.13	0.02	2.00	0.05

$\delta^{13}C_{ARA}$

ANOVA:	Sum Sq	Mean Sq	NumDF	DenDF	F	p	
	3.24	1.08	3.00	4.23	0.56	0.67	
Random effect:	Variance	SD					
tank	11.36	3.37					
residual	1.91	1.38					
				LSM differences			
treatment	ß	SE	DF	*t*-value	Lower CI	Upper CI	p
benthic - baseline	2.10	4.29	4	0.50	-9.53	13.78	0.60
benthic - mixed	3.80	3.11	5	1.22	-4.36	11.93	0.30
benthic - terrestrial	3.80	3.55	5	1.06	-5.69	13.20	0.30
baseline - mixed	1.70	3.85	4	0.43	-9.10	12.42	0.70
baseline - terrestrial	1.60	4.21	4	0.39	-10.16	13.41	0.70
mixed - terrestrial	0.00	3.01	4	-0.01	-8.31	8.25	1.00

$\delta^{13}C_{EPA}$

ANOVA:	Sum Sq	Mean Sq	NumDF	DenDF	F	p	
	10.94	3.65	3.00	3.35	0.65	0.63	
Random effect:	Variance	SD					
tank	1.05	1.02					
residual	5.58	2.36					
				LSM differences			
treatment	ß	SE	DF	*t*-value	Lower CI	Upper CI	p
benthic - baseline	-1.40	2.34	5	-0.59	-7.52	4.77	0.60
benthic - mixed	0.80	2.04	10	0.42	-3.70	5.40	0.70
benthic - terrestrial	-0.60	2.18	8	-0.26	-5.63	4.48	0.80
baseline - mixed	2.20	1.73	2	1.28	-5.73	10.18	0.30
baseline - terrestrial	0.80	1.90	2	0.42	-7.67	9.28	0.70
mixed - terrestrial	-1.40	1.52	3	-0.94	-6.14	3.30	0.40

$\delta^{13}C_{DHA}$

ANOVA:	Sum Sq	Mean Sq	NumDF	DenDF	F	p	
	19.51	6.50	3.00	4.69	1.94	0.25	
Random effect:	Variance	SD					
tank	46.29	6.80					
residual	3.36	1.83					
			LSM differences				
treatment	ß	SE	DF	*t*-value	Lower CI	Upper CI	p
benthic - baseline	-15.40	8.47	5	-1.81	-37.60	6.87	0.10
benthic - mixed	-12.50	6.06	5	-2.05	-28.10	3.21	0.10
benthic - terrestrial	-14.10	6.97	5	-2.02	-32.20	4.05	0.10
baseline - mixed	2.90	7.67	5	0.38	-17.40	23.26	0.70
baseline - terrestrial	1.30	8.41	5	0.15	-21.00	23.60	0.90
mixed - terrestrial	-1.60	5.97	5	-0.27	-17.40	14.13	0.80

Bulk $\delta^{13}C$

ANOVA:	Sum Sq	Mean Sq	NumDF	DenDF	F	p	
	3.66	1.22	3.00	5.14	1.65	0.29	
Random effect:	Variance	SD					
tank	0.34	0.58					
residual	0.74	0.86					
			LSM differences				
treatment	ß	SE	DF	*t*-value	Lower CI	Upper CI	p
benthic - baseline	-1.20	1.02	5.80	-1.19	-3.71	1.30	0.28
benthic - mixed	-1.50	0.84	9.80	-1.75	-3.34	0.41	0.11
benthic - terrestrial	-2.00	0.92	8.20	-2.19	-4.10	0.10	0.06
baseline - mixed	-0.30	0.81	3.40	-0.32	-2.69	2.16	0.76
baseline - terrestrial	-0.80	0.89	3.40	-0.90	-3.45	1.85	0.43
mixed - terrestrial	-0.50	0.68	4.60	-0.79	-2.33	1.25	0.47

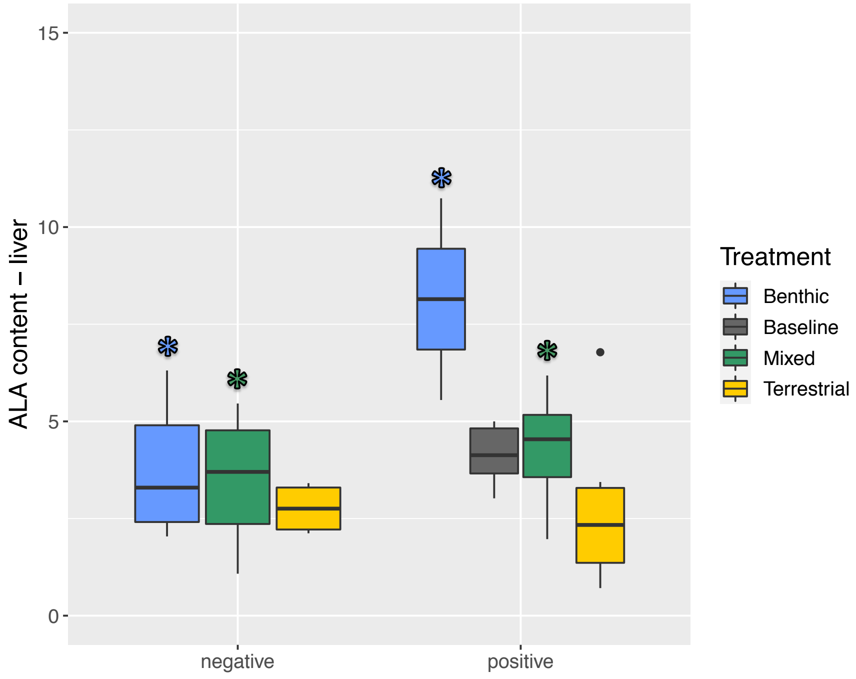

Figure S1. Differences in ALA content between fish with negative and positive weight change during the experiment for each treatment group. Blue indicates benthic treatment, green indicates mixed treatment, and yellow indicates terrestrial treatment. Grey indicates fish with baseline diet. Colored asterisks denote significant differences ($\alpha = 0.05$) between weight groups within each diet treatment from a Welch two-sample t-test with a Hommel correction, e.g., the blue asterisk denotes a significant difference between weight groups in the benthic treatment.

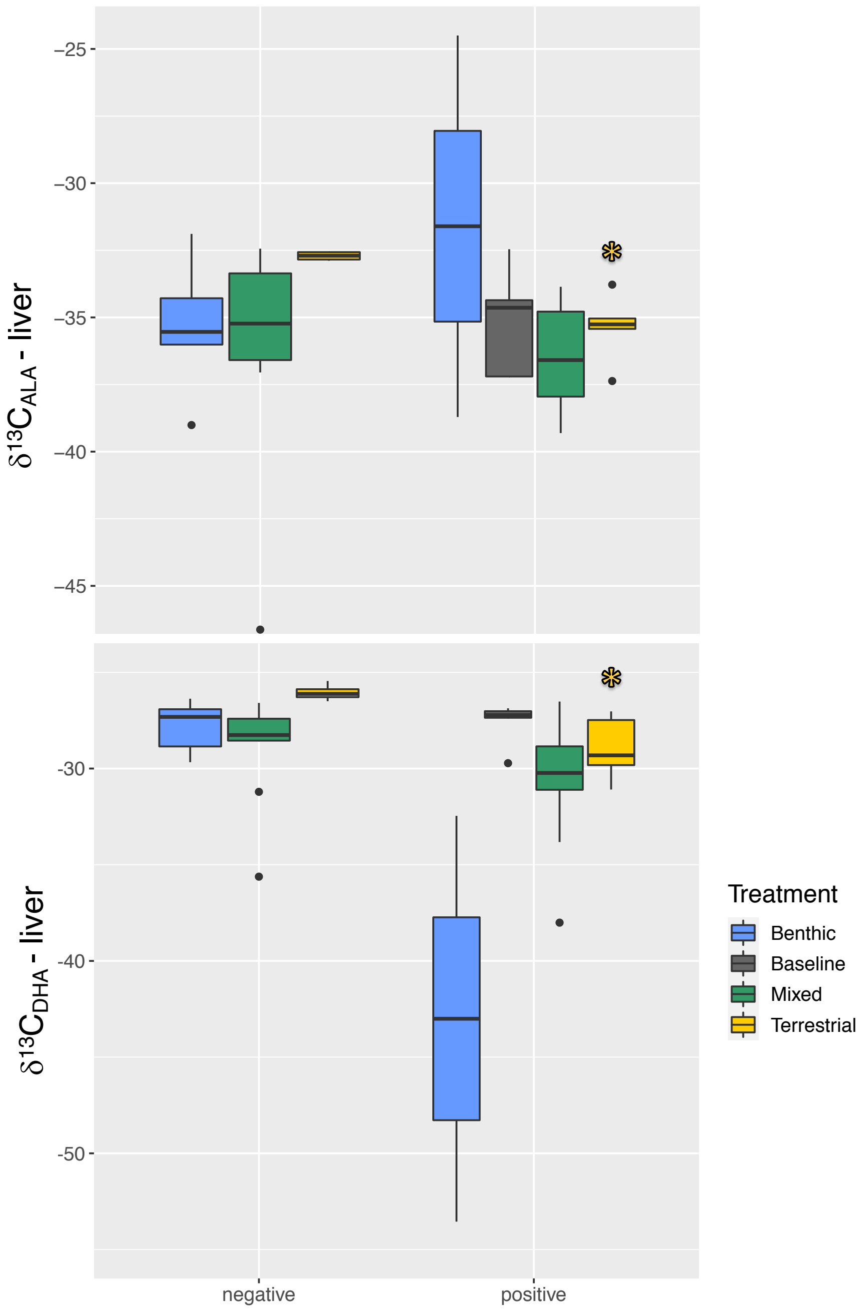

Figure S2. Differences in ALA and DHA δ^{13}C values between fish with negative and positive weight change during the experiment for each treatment group. Blue indicates benthic treatment, green indicates mixed treatment, and yellow indicates terrestrial treatment. Grey indicates fish with baseline diet. Colored asterisks denote significant differences ($\alpha = 0.05$) between weight groups within each diet treatment from a Welch two-sample t-test with a Hommel correction.
